# Dataset of numerical study of the impact of two-step injection (direct and port) of hydrogen-enriched and natural gas mixtures on engine efficiency and exhaust emissions

**DOI:** 10.1016/j.dib.2024.110894

**Published:** 2024-09-03

**Authors:** Javad Zareei, Sabir Tagelsir Hassan widatalla, John William Grimaldo Guerrero, Navruzbek Shavkatov, Qusay Rasheed Al-amir

**Affiliations:** a Department of Biosystem Engineering, Ferdowsi University of Mashhad, Iran; b Department of Mathematics, Faculty of Science, University of Tabuk, P.O.Box741, Tabuk 71491, Saudi Arabia; c Department of Energy, Universidad de la Costa, Barranquilla, Colombia; d The Department of Corporate Finance and Securities, Tashkent State University of Economics, Tashkent, Uzbekistan; e College of Engineering and Technologies, Mechanical Power Technical Engineering Department, Al-Mustaqbal University, 51001 Babylon, Iraq

**Keywords:** Two step injection, Hydrogen, Natural gas, Performance, Emissions

## Abstract

A study was conducted to examine the effects of two-step fuel injection on a modified four-cylinder engine that was converted from port to direct injection. The primary fuel source utilized was hydrogen-enriched compressed natural gas (HCNG), which replaced the conventional gasoline. In the initial phase of the procedure, compressed natural gas (CNG) was introduced into the intake manifold at a concentration of 10 % by mass, relative to the total fuel mixture. The remaining 90 % of the fuel consisted of HCNG, which was injected directly into the cylinders. The injection of compressed natural gas (CNG) commenced at 160° before top dead center (BTDC) with a 20° stroke duration. The HCNG fuel was injected in a two-step process. In the initial phase, HCNG was injected at 130° BTDC with a 50° stroke duration, with a stepwise increase from 0 % to 40 %.

The study employed AVL software for the assessment of engine performance, efficiency, fuel consumption, and exhaust emissions. The data collected indicated that the injection of a 30 % HCNG blend resulted in an increase in brake power, brake thermal efficiency, and in-cylinder pressure (from 8 % to 13.64 %), as well as a reduction in specific fuel consumption (by 18 %). This improvement was attributed to an increase in flame propagation speed within the combustion chamber. Additionally, the percentage of excess hydrogen was found to decrease, resulting in a reduction of carbon monoxide and unburned hydrocarbons by up to 14 % due to complete combustion. However, NOx increased due to the rise in exhaust temperature.

Specifications Table

**Table:** 

Subject	Automotive Engineering and Renewable Energy
Specific subject area	Numerical Study of the Impact of Two-Step Injection (Direct and Port) of Hydrogen-Enriched and Natural Gas Mixtures.
Data format	Raw, Analysed, Filtered
Type of data	Table, Image, Chart, Graph, Figure
Data collection	In order to collect data, a single-cylinder CNG-DI engine is equipped with two exhaust valves and two intake valves. In order to gain insight into the patterns and behavior of turbulence, tumble, and swirl intensity fields within the cylinder, two distinct piston shapes were subjected to testing. Ultimately, the bowl piston was determined to be the optimal choice for the combustion process. Prior to this selection, an analysis of heat transfer, turbulence characteristics, and fuel mixture preparation was conducted for both piston shapes. Subsequently, the piston surface data was transformed into a finite volume mesh for CFD analysis. The Gambit software was employed for the generation of the hexahedral grid and the computation of the computational mesh. The finite volume method was employed in the CFD calculations. The three-dimensional models incorporated nodes, faces, volumes, and numerical values for the desired quantities at specific node positions. A moving mesh boundary algorithm was employed for CFD simulations of combustion. Each process was conducted with the moving mesh algorithm and a specified boundary layer at each engine cycle step. A mesh was generated for each surface using CAD software. To enhance the quality of the mesh, a hybrid type of boundary (tetrahedral, hexahedral, pyramidal, and wedge elements) was employed. The computational domain encompassed the four valves, the intake port, the piston bowl, and the cylinder head. The number of cells in the TDC was 222,315, whereas the BDC exhibited a variable number of cells, ranging from 719,019 to 918,428. This was compared to the 2007 study by Wendy et al., which had 90,000 cells in the TDC and 180,000–200,000 cells in the BDC. In order to analyze the effect of engine speed, injection, and ignition timing on performance, a computational fluid dynamics (CFD) calculation was performed. The crank angle degree was defined in the range of 0–720° by establishing the initial pressure and temperature values. The simulation was terminated at top dead center, which coincides with the opening of the exhaust valve. As the engine speed increased, the injection and ignition timings were modified accordingly. It has been demonstrated that a tolerable engine run necessitates advance/retard adjustments.
Data source location	All data were collected in 2021 by means of the AVL Fire software, and the static models were collected at Ferdowsi University of Mashhad and De la Costa University. The data were collected at the Department of Biosystem Engineering, Ferdowsi University of Mashhad, Iran.
Data accessibility	Repository name: Mendeley Data Data identification number: 10.17632/wvg9jg8j8w.1 Direct URL to data: https://data.mendeley.com/datasets/wvg9jg8j8w/1
Related research article	Zareei J, Rohani A, Mazari F, Vladimirovana M, Numerical investigation of the effect of two-step injection (direct and port injection) of hydrogen blending and natural gas on engine performance and exhaust gas emissions, Energy Journal. 2021.

## 1 Value of the Data

The data obtained from this study on the Dataset of Numerical Study of the Impact of Two-Step Injection (Direct and Port) of Hydrogen-Enriched and Natural Gas Mixtures on Engine Efficiency and Exhaust Emissions is of significant value for a number of reasons.

• The dataset is of significant value due to its comprehensive testing of multiple engine operating parameters, including engine power, fuel consumption, and emissions. The dataset comprises data on various fuel blends and injection timings, which may prove useful for further engine optimization studies.
• The dataset can be utilized as a research reference, as it offers a comprehensive assessment of the impact of two-stage fuel injection and hydrogen-enriched fuels on engine performance and emissions. The dataset can be employed to either corroborate or challenge existing literature on the subject, thereby providing insights for further research.
• This study addresses the impact of two-stage fuel injection and hydrogen-enriched fuels on engine performance and emissions. The dataset is centered on the impact of disparate injection timings and fuel blends on engine operation, which is crucial for optimizing engine performance and minimizing emissions.
• The dataset may be utilized for the development and validation of engine control and evaluation methods, including the creation of simulation models and the establishment of test protocols. The comprehensive testing conducted within this dataset serves as a valuable reference point for the development and assessment of models in the domain of engine optimization.
• The dataset may be utilized for the development and validation of engine control and evaluation methods, including the creation of simulation models and the establishment of test protocols. The comprehensive testing conducted within this dataset serves as a valuable reference point for the development and assessment of models in the domain of engine optimization.
• The dataset serves to validate the simulation model and test protocols utilized in this study, thereby instilling confidence in the model results and ensuring the accuracy of said results.

## 2 Background

The development of cleaner and non-fossil fuels, such as hydrogen, for internal combustion engines has become a significant area of interest in recent years [1]. One approach is the use of hydrogen-enriched compressed natural gas (HCNG), which has the potential to enhance engine performance and reduce exhaust emissions [2]. The utilization of HCNG has been demonstrated to diminish unburned hydrocarbons and carbon monoxide, augment engine performance, and reduce fuel consumption [3,4]. Furthermore, the incorporation of hydrogen into natural gas has been demonstrated to enhance engine efficiency and mitigate emissions. This paper presents a discussion of the research and findings related to the use of HCNG and hydrogen in internal combustion engines. Furthermore, this subject provides specific percentage recommendations for the use of hydrogen in natural gas blends and discusses how different injection timings and fuel blends can affect engine performance [5,6]. The utilisation of simulation software, such as AVL Fire software, has been demonstrated to yield precise outcomes in the examination of the impact of diverse fuel blends on engine performance [7]. Furthermore, the authors emphasize that the incorporation of hydrogen can effectively reduce emissions without necessitating alterations to the combustion chamber or engine design. Furthermore, researchers have demonstrated that the incorporation of 15–75 % hydrogen in the injectable fuel can result in thermal loss and enhanced combustion outcomes. Initially, this leads to a reduction in soot pollutants, an increase in NOx, and an improvement in thermal efficiency, while simultaneously increasing the percentage of hydrogen [[8], [9], [10], [11]]. Another study by Kakoee et al. [12] demonstrated that the addition of 30 % hydrogen to natural gas resulted in a 29.8 % reduction in the amount of UHC and a 35.5 % reduction in the amount of CO. Another study has demonstrated that the addition of 10 % hydrogen enrichment to natural gas in diesel engines results in fuel consumption of 236 g/kWh at 70 % engine load [13]. In a separate study, the numerical results of combining hydrogen fuel with natural gas in a direct injection (DI) engine demonstrated that a 30 % hydrogen blend increased engine torque and power output while simultaneously reducing CO emissions when the ignition timing was advanced [14,15]. Moreover, the advance of ignition timing is up to 21° before top dead center, while the equivalent of 30 % hydrogen in blending optimizes engine performance [16]. Furthermore, the reduction in emissions is achieved without any modifications to the combustion chamber or engine design changes [17]. Other researchers have determined that the maximum braking torque of a single-cylinder port injection engine fueled with HCNG is inferior to that of the base natural gas engine due to the lower knock limit [18,19].

The objective of this paper is to analyse the impact of a two-step fuel injection on a four-cylinder engine that has been converted from port to direct injection. In the initial phase, 10 % of the CNG is added by mass and injected into the air manifold as a pre-mixed fuel. In the second step, HCNG fuel is directly injected into the engine cylinders in steps of 10 %, 20 %, 30 %, and 40 %. Previous research has focused on one-step injection procedures. This paper presents a new method of two-step fuel injection, with the aim of improving engine performance and reducing exhaust emissions. The objective is to identify the optimal HCNG fuel injection value, which will deliver the best engine performance and the lowest possible exhaust emissions. AVL software was employed to assess engine performance, efficiency, fuel consumption and exhaust emissions. The data collected revealed that injecting a 30 % HCNG blend led to improvements in brake power, brake thermal efficiency, and in-cylinder pressure, as well as specific fuel consumption. The reduction in the percentage of excess hydrogen resulted in a decrease in carbon monoxide and unburned hydrocarbons by up to 14 % due to complete combustion. However, there was an increase in NOx due to an increase in exhaust gas temperature. In conclusion, two-step fuel injection represents an innovative approach that enhances engine performance and reduces exhaust emissions.

## 3 Data Description

The present study examines the impact of two-step fuel injection on a four-cylinder engine that has undergone a modification from port injection to direct injection. A hydrogen-enriched compressed natural gas (HCNG) blend has been employed as an alternative to gasoline. In the initial phase, compressed natural gas (CNG) is introduced at a ratio of 10 % of the total fuel mass fraction into the air manifold, while the remaining 90 % of the fuel is hydrogen-enriched compressed natural gas (HCNG), which is injected directly into the cylinder. The primary injection of CNG occurred at 160° BTDC with a 20° stroke duration. The HCNG fuel was injected at 130° BTDC with a 50° stroke duration, with the injection of fuel occurring in increments of 10 % from 0 % to 40 %.

The investigation has employed AVL software for the purpose of scrutinizing engine performance and efficiency, fuel consumption, and exhaust emissions. In this study, the spark ignition (AKTIM model) and auto-ignition were modelled at the origin of the knock phenomenon. Both models are embedded within an ECFM3Z model framework. The ECFM3Z model is employed for the purpose of modeling classical combustion processes. The initial objective of the present computational study was to identify a laminar flame speed correlation that would be optimally suited to represent the combined combustion of methane and hydrogen for a range of volume ratios. The majority of associations pertaining to the combustion of light hydrocarbons are primarily founded upon the original formulation proposed by Methgalchi and Keck for mixtures of air with propane, methanol, isooctane, or indolence [20,21].

The laminar flame speeds are obtained by critically comparing and analyzing the measurements, thereby developing an accuracy in empirical correlations to obtain laminar flame speeds for equivalent ratio and unburned mixture temperature.

For the purposes of the CFD simulation, the engine operating conditions were selected at speeds of 2000, 4000, and 6000 rpm.

In the CFD calculation, the crank angle degree was set to a range of 0–720° CA, with each engine cycle defined by the initial pressure and temperature values. The simulation was concluded at the top dead center, which coincides with the opening of the exhaust valves. As the engine speed increased, the injection and ignition timings were modified accordingly.

### 3.1 Data set -grid generation for combustion chamber

**Fig. 1:**
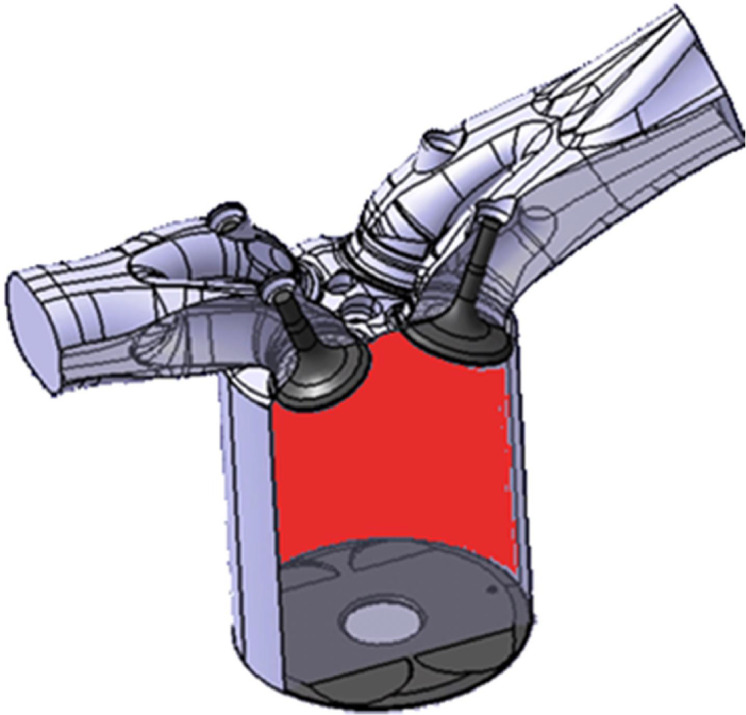
Designed model of a single-cylinder natural gas direct-injection engine.

**Fig. 2:**
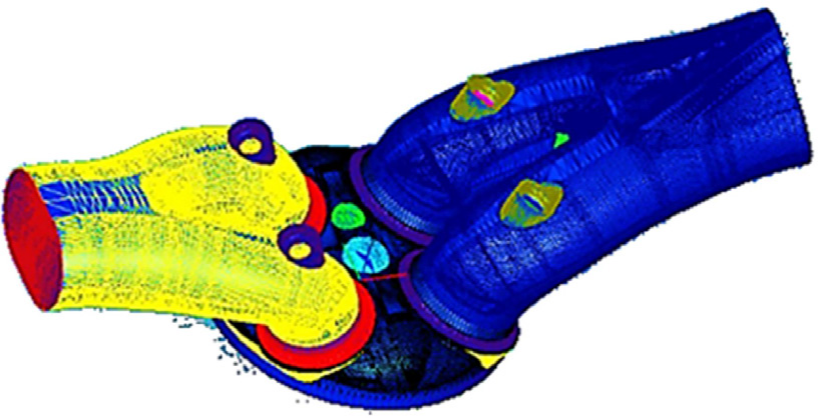
The computational domain of the engine model.

In this study, the moving mesh boundary algorithm was employed to conduct computational fluid dynamics (CFD) simulations of the combustion process in a four-cylinder piston engine. The moving mesh algorithm and CAD software were employed to generate mesh levels that corresponded to the crank angle, geometries, and mesh levels in each engine cycle step. The number of mesh cells was calculated based on the initial geometry of all parts and subsequently selected. The subsequent phase of the process entailed the examination of potential computational inconsistencies within the advanced model through the utilization of CFD simulations, necessitating the pre-processing stage. The pre-processor tools were employed to generate the mesh, and the GAMBIT software was utilized to define the boundary types of the domain. A hybrid mesh type was employed to optimize the mesh quality, comprising a total of 222,315 cells within the TDC range and approximately 719,019 to 918,428 cells (in comparison to the engine model presented by Wendy et al. (2007), which comprised 90,000 cells within the TDC range and approximately 180,000 to 200,000 cells within the BDC range). Additionally, a greater number of cells were incorporated into the piston bowl and cylinder head within the BDC. In the present study, a hybrid mesh was employed to enhance the quality of the domain mesh. As illustrated in Figs. 1 and 2, the computational domain encompasses four valves, the intake port, the piston bowl, and the cylinder head.

**Fig. 3:**
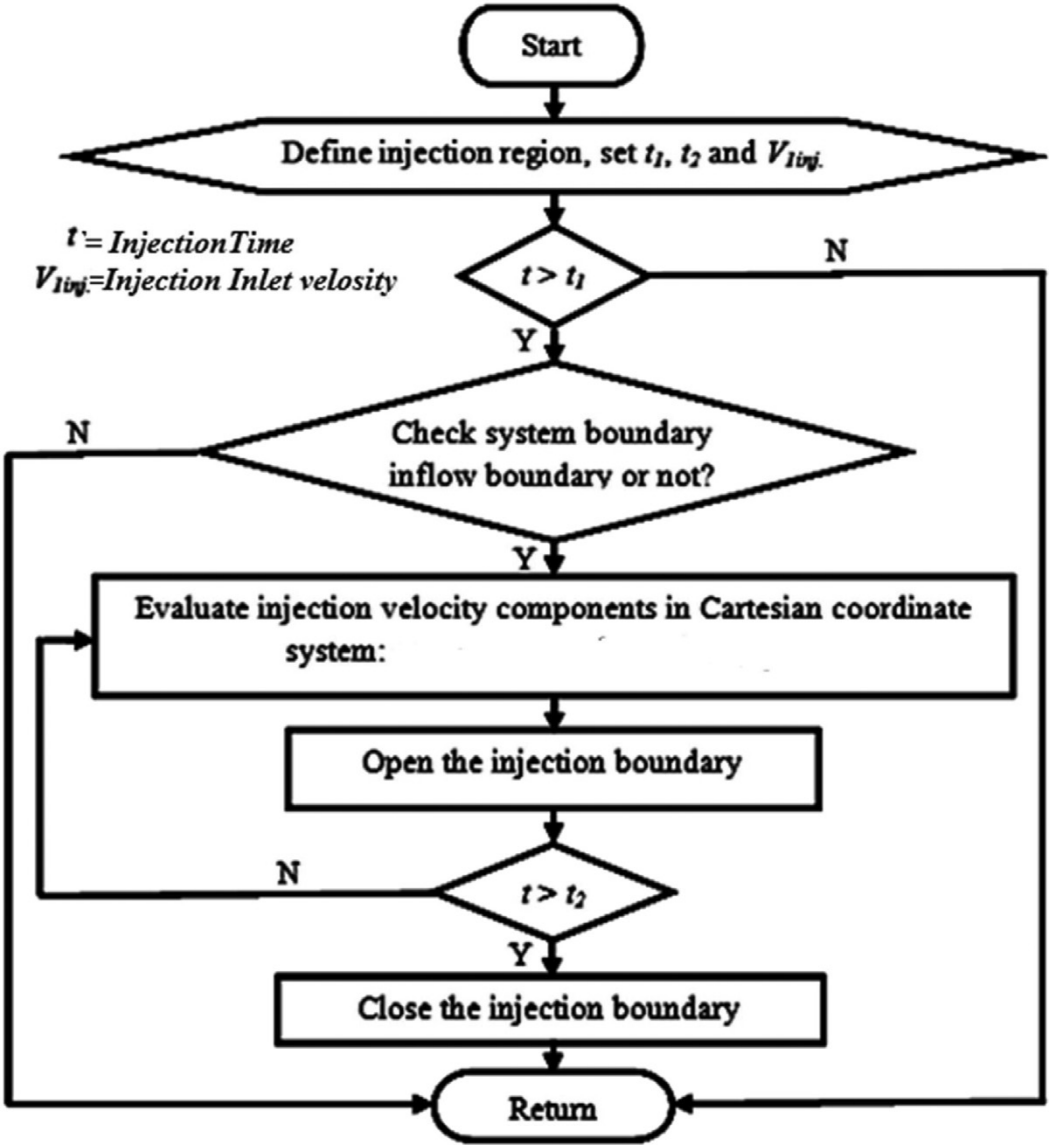
Flowchart for the injection program.

Fig. 3 shows a flowchart detailing the fuel injection program within the combustion chamber of a gasolin engine. This program has been integrated with the main AVL Fire code to complete the multidimensional model. The Chemkin mechanism simulates the fuel injection process as a boundary condition, setting the inlet velocity based on values obtained from experimental tests at the onset of fuel injection through the velocity open boundary.

### 3.2 Statistical method

An analysis of variance (ANOVA) and Tukeyʼs tests are statistical methods employed to compare the means of multiple groups. In the present study, we employed the use of ANOVA to analyze the effects of alterations in fuel mixing percentages on the ignition delay of dual-fuel gasolin engines. ANOVA is a parametric method that assumes that the data is normally distributed. The ANOVA test provides a basis for comparing the means of two or more groups, and Tukeyʼs test is a method of multiple comparisons used with ANOVA to determine which particular means differ from one another. The deployment of these methodologies was predicated on the assumption that the data exhibited a normal distribution. Furthermore, ANOVA and Tukeyʼs tests have been extensively employed in scientific research and are regarded as reliable and valid techniques for data analysis.

Randomized design (RD) was implemented for observing and comparing the effect of the fuel mix on engine power and exhaust emissions at 3 different RPM speeds (2000, 4000, and 6000). The findings of the analysis of variance (ANOVA) through the F-statistic were employed to evaluate the null hypothesis (H_0_), suggesting that the average engine power remains constant across the 5 different hydrogen usage levels (Eq. 1).

(1) $$H_{0}:\mu_{0\%}=\mu_{10\%}=\mu_{20\%}=\mu_{30\%}=\mu_{40\%}$$

If the *p*-value is less than 1 % or 5 %, then the influence of hydrogen on the engine performance parameters is significant and H0 is rejected. The p-value for the ANVOA of hydrogen blending is less than 0.02, indicating that the mean differences between CNG and hydrogen fuel are statistically significant. Pairwise comparisons and performance parameters (brake power, brake thermal efficiency, specific fuel consumption, in-cylinder pressure, unburned hydrocarbons, carbon monoxide, and NOx) were made by comparing Tukeyʼs means at 1 % significance level (*p*-value ≤ 0.01). Therefore, the effect of hydrogen application on engine performance changes can be compared statistically. ANOVA and Tukeyʼs test were performed using Minitab software, version 19.

The statically results showed that an increase in engine speed results in an enhancement of engine power. However, an escalation in the proportion of hydrogen within the fuel blend gives rise to an augmentation of flame speed, reaching a maximum of 30 % HCNG, after which a decline is observed. The statically findings of the present study indicate that the injection of hydrogen into the air manifold as a pre-mixed fuel, followed by the direct injection of the main fuel into the engine, can result in enhanced performance in terms of engine torque, brake thermal efficiency, and brake-specific fuel consumption. The optimal hydrogen content for attaining maximum engine torque has been determined to be approximately 30 %. Additionally, the study demonstrates that the maximum combustion chamber pressure rises in conjunction with an increase in engine speed and hydrogen content. Furthermore, the data indicates that a fuel blend containing 40 % hydrogen produces the most significant pressure compared to other levels of hydrogen. The study also suggests that the addition of hydrogen to the fuel mixture results in a notable increase in the maximum heat release rate at a level of 1 %. Additionally, the angular location of maximum pressure plays a crucial role in regulating engine output pollutants.

Additionally, the statistical analysis indicates that an increase in the percentage of hydrogen in the fuel blend results in a reduction in the emission of unburned hydrocarbons and CO, while concurrently leading to an increase in NOx emission in comparison to natural gas. Nevertheless, at elevated engine speeds, the NOx emission with hydrogen fuel is greater than that with natural gas, due to the higher temperatures at higher speeds. It is crucial to highlight that the utilization of a pre-mixture of fuel with air in the initial stage of injection and injection of fuel directly into the combustion chamber in the subsequent stage can enhance the fuel blend and facilitate complete combustion. The study also corroborates that hydrogen fuel is devoid of carbon and has been observed to reduce CO₂ by 14 % in comparison to natural gas.

## 4 Experimental Design, Materials and Methods

This study employs a two-stage compression ignition system in a four-cylinder engine with direct injection of compressed natural gas and a blended fuel mixture of natural gas and hydrogen. The hydrogen blend was utilized in varying ratios, including 10 %, 20 %, 30 %, and 40 %. The investigation examined the impact of the fuel blend on engine performance and exhaust emissions at three distinct engine speeds (2000, 4000, and 6000 RPM). CFD simulations were used with selected engine operating conditions, including intake temperature, injection timing, injection duration, and spark timing. The engine operating conditions are listed in Table 1.

**Table 1:** Specification of the compressed natural gas direct injection engine.

Engine parameter	Value	Unit	Engine parameter	Value	Unit
Maximum rated power	82/6000	kW/rpm	Intake valve opening	12	bTDC
Maximum rated torque	148/4000	Nm/rpm	Intake valve closing	48	aBDC
Stroke	84	Mm	Exhaust valve opening	45	bBDC
Connecting rod length	131	Mm	Exhaust valve closing	10	aTDC
Crank radius	44	mm	Maximum intake valve lift	8.1	mm
Compression ratio	14	–	Maximum exhaust valve lift	7.5	mm
Fuel Displacement	1597 CM^3^ Fuel: CNG+ Hydrogen				

In the CFD calculations, the crank angle degree was defined from 0° to 720 °CA, and the engine cycle events were specified by setting initial pressure and temperature values. The simulation ended at top dead center when the exhaust valves opened. Injection and ignition timings were adjusted as engine speed and mixture increased. Research shows that it is acceptable to adjust spark advance and retard to improve engine performance. In this study, the engine speed was incrementally increased from 2000 to 6000 rpm as the combustion process was advanced. Injection timing started at 130 °CA at 2000 rpm and increased by 40° with each run. Similarly, ignition timing adjustments were made, with an increase of approximately 4° at each stage. Respectively in all engine speeds, as the speed increases, the combustion timing and injection timing are considered as an advance, which the amount varies according to the engine design. It is also assumed that the fuel-air mixture is perfectly mixed, although this may not be the case in real-world scenarios. The ignition delay is treated as a constant parameter, and its dependence on engine speed, injection timing, and fuel mixing percentage may not be fully considered. Furthermore, the model may not account for the effect of humidity and other environmental factors on the combustion process. The baseline operating conditions of the selected engine are listed in Table 2.

**Table 2:** Baseline engine operating conditions [22].

Engine parameters and unit	Value		
Engine Speed (rpm)	2000	4000	6000
CNG mass(mg)	5.2	5.2	5.2
Equivalence Ratio	1.0	1.0	1.0
Intake Port Temperature (K)	305	305	306
Intake Port Pressure (bar)	1.04	1.02	0.9
Start of Injection Timing (bTDC)	130	170	210
End of Injection Timing (bTDC)	80	120	160
Spark Ignition Timing (bTDC)	19	23	28
Injection pressure(bar)	20	20	20

Table 3 shows the energy and mass composition of all fuels used in the study. Table 4 shows some properties of hydrogen and methane (which is very close to the properties of natural gas) fuel under stoichiometric condition. In Table 3, the values are obtained from the thermodynamic table by changing the percentage of composition and further using Eq. (1) to obtain these values.

**Table 3:** Energy and mass composition of H_2_-NG fuel.

Unit	CNG	HCNG10	HCNG20	HCNG30	HCNG40
H_2_(% Mass)	0	1.21	2.69	4.52	6.72
H_2_(% energy)	0	3.09	6.68	10.49	15.59
LHV(MJ/Kg)	46.28	47.17	48.26	49.61	51.41
LHV stoich. Mixture (MJ/NM^3^)	3.376	3.359	3.353	3.349	3.344
CNG mass(mg)	5.2	5.13708	5.06012	4.96496	4.855
Hydrogen mass(mg)	0	0.06292	0.13988	0.23504	0.3450

**Table 4:** Properties of hydrogen and methane fuel under stoichiometric condition [23].

Properties	Hydrogen	Methane	Unit
Flammability limits	4–75	5–15	Vol. %
Minimum ignition energy	0.02	0.29	MJ
Flame temperature	2045	1875	°C
Autoignition temperature	585	540	°C
Diffusion coefficient	0.61	0.20	10^−3^ m^2^/s
Maximum velocity of flame	3.46	0.43	m/s
Density	0.65	0.08	kg/m^3^

In order to calculate the quantity, Table 3 should be solved in the equation of the combustion.

To obtain exhaust emissions, it is necessary to couple the Chemkin and AVL Fire software. When the Chemkin code and AVL fire software are coupled, an integrated simulation model can be created to perform chemical modeling and fire simulation. The Chemkin code is used to simulate the chemical processes occurring within the combustion chamber of a gasolin engine, while the AVL fire software is used to simulate the fire dynamics of the exhaust gases.

To obtain exhaust results in a gasolin engine, the first step is to conduct a chemical simulation of the combustion process using Chemkin. The Chemkin model takes into account various chemical species, such as oxygen, carbon monoxide, nitrogen oxides, and hydrocarbons, as well as their reactions during combustion. By simulating the chemical processes, we can determine the engine performance parameters, such as brake horsepower, brake thermal efficiency, specific fuel consumption, and exhaust emissions.

Upon completion of the chemical simulation, the exhaust gases are then simulated using AVL fire software. The simulation provides valuable insight into the heat release rate, temperature distribution, and location of hot spots within the exhaust system. The results of the simulation can then be used to calculate the maximum temperature and the pressure drop in the exhaust system.

The integration of the Chemkin code and AVL fire software allows for the creation of an integrated simulation model, which provides a more comprehensive understanding of the combustion process and its impact on the exhaust gases in a gasolin engine. The simulation results can be leveraged to optimize engine design, refine emission control strategies, and enhance the overall performance of gasolin engines.

While numerical solutions in computational fluid dynamics offer impressive problem-solving capabilities, they require validation for accuracy. The validation process depends on the type of problem and the solution conditions. For combustion problems, validation is often done in two steps. The first step is to optimize the mesh values, and the second step is to compare the numerical solution results with experimental data. In this research, the first step was to find an optimal solution field mesh and then to compare the numerical results with experimental data [24].

**Fig. 4:**
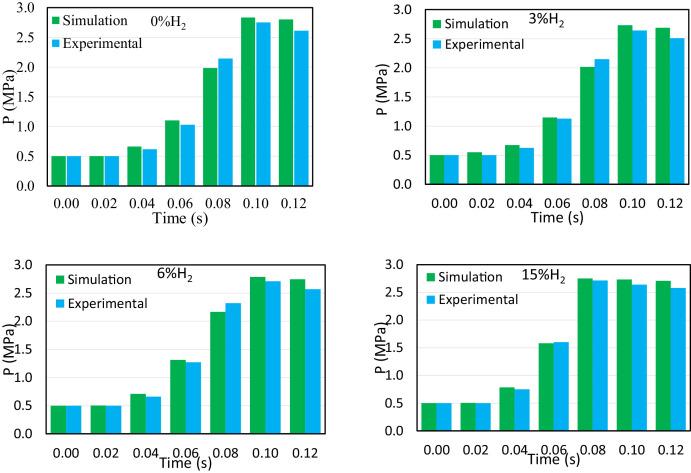
Comparison of combustion chamber pressure between experimental and simulation data in four modes (0, 3, 6, and 15 %) of hydrogen in the fuel mixture for different times.

Fig. 4 illustrates a comparison of combustion chamber pressure between experimental and simulated data for four distinct hydrogen blending modes, namely 0 %, 3 %, 6 %, and 15 %. The results demonstrate a high degree of similarity between the pressure change trends observed in both the simulations and the experiments. Accordingly, the discrepancy between the two methods is deemed to be within an acceptable range, with a difference of only 3.82 % between the pressures observed.

## Limitations

The research is subject to certain constraints pertaining to the composition of the fuel utilized, which is a blend of hydrogen and compressed natural gas (CNG) prior to injection. This has an impact on the combustion performance. Furthermore, limitations exist with regard to the design, injection timing, and compression ratio. The compression ratio is of critical importance with regard to the performance of fuels such as hydrogen and compressed natural gas (CNG). In order to address these limitations, it is essential to consider them during the design phase of the engine in order to mitigate potential issues and optimize performance with improved efficiency.

## Ethics Statement

Authors have read and agree to abide by the ethical requirements for publication in Data in Brief and confirm that the current work does not involve human subjects, animal testing, or data collected from social media platforms.

## CRediT Author Statement

**Javad Zareei:** Writing – review & editing, Writing – original draft, Visualization, Validation, Supervision, Software, Resources, Project administration, Methodology, Investigation, Funding acquisition, Formal analysis, Data curation, Conceptualization. **Sabir Tagelsir Hassan widatalla**: Writing – review & editing, Writing – original draft, Resources, Investigation, **John William Grimaldo Guerrero**: Writing – review & editing, Writing – original draft, Conceptualization, **Navruzbek Shavkatov:** Writing – review & editing, Writing – original draft, Conceptualization, **Qusay Rasheed Al-amir:** Writing – review & editing, Writing – original draft, Conceptualization.

## Data Availability

Dataset of Numerical Study of the Impact of Two-Step Injection (Direct and Port) of Hydrogen-Enriched and Natural Gas Mixtures on Engine Efficiency and Exhaust Emissions (Original data) (Mendeley Data).
